# The Relative Associations of Body Image Dissatisfaction among Psychiatric Out-Patients in Singapore

**DOI:** 10.3390/ijerph16245162

**Published:** 2019-12-17

**Authors:** Pratika Satghare, Mithila Valli Mahesh, Edimansyah Abdin, Siow Ann Chong, Mythily Subramaniam

**Affiliations:** Research Division, Institute of Mental Health Singapore, Singapore 539747, Singapore; mithilam91@gmail.com (M.V.M.); edimansyah_abdin@imh.com.sg (E.A.); siow_ann_chong@imh.com.sg (S.A.C.); mythily@imh.com.sg (M.S.)

**Keywords:** body image dissatisfaction, mental illness, psychological distress, Singapore

## Abstract

*Background*: Adults with body image dissatisfaction (BID) are more likely to be depressed, anxious, and suicidal when compared to those without intense dissatisfaction over their appearance. The current study aimed to estimate the prevalence and factors associated with BID among out-patients with mental illness in Singapore. *Methods*: Data was collected from 310 psychiatric out-patients using a self-administered questionnaire. Measurements used were socio-demographic characteristics, Body Mass Index scores, Body Shape Questionnaire, Binge Eating Scale, Eating Attitudes Test, Beck’s Depression Inventory, Beck’s Anxiety Inventory and Alcohol Use Disorders Identification Test. *Results*: A prevalence of 30.9% of BID was established among psychiatric out-patients in Singapore. Being female, having higher BMI scores, binge eating behavior, eating disorders, and those diagnosed with depression were positively associated with BID. *Conclusion*: BID is prevalent among those with psychiatric illnesses which could lead to a higher degree of psychological distress and the emergence of eating disorders.

## 1. Introduction

Body image dissatisfaction (BID) is defined as a negative subjective evaluation of the weight and shape of one’s own body [1]. Biological, interpersonal, and sociocultural factors, particularly media exposure as it is ubiquitous and powerful, play an influential role in the development of body image dissatisfaction among both men and women [2]. Previous literature has reported prevalence rates of body image dissatisfaction ranging from 19.5% to 77% among the general adolescent population [3,4,5,6,7]. According to Steg et al. [8], poor body image could be associated with self-esteem issues, depression, anxiety, and disordered eating behaviors. A number of studies have reported that BID is a risk factor for the development of depression [9,10,11,12], suicidal ideation [13,14], and social anxiety [15]. 

A population study among American college men displayed substantial body dissatisfaction that was closely associated with depression, use of performance-enhancing substances, and low self-esteem [16]. Previous research suggested that a causal pathway occurs between perceived obesity status and depression through weight stigmatization and body dissatisfaction [17,18]. Sertoz et al.’s study among somatising patients has found high comorbidity of depression and anxiety due to dissatisfaction with body appearance [19], whereas another study perceived BID to be significantly related with psychological variables, especially anxiety due to weakened sense of self experienced by an individual [20]. Weight gain which is often observed in patients with schizophrenia leads to both BID and negative body image leading to decrease in quality of life and self-esteem [21]. In a study of 167 patients with schizophrenia spectrum disorders, Oh et al. [22] found a significant association between negative body image and low self-esteem however, body mass index was not associated with body image. 

The proliferation of Western media in Eastern cultures may be a contributing factor to shifts in sociocultural values in non-Western communities [23]. In Asian societies, especially among adolescent and young adults, physical appearance, particularly the drive for slenderness, is seen prominently among females as compared to males [24,25]. A study among young Asian males seeking treatment for eating disorder expressed significant fear of BID accompanied by fat phobia [26]. Strong implicit anti-fat bias internalization was reported as a valid predictor of weight-related behavioral concerns leading to body image trepidations among Asian female students [27]. Thus, BID is not only prevalent in developed Western countries but also is increasingly seen in developing non-Western countries [28]. 

The emotional construct of BID has lately gained attention in scientific and clinical research due to it being a diagnostic trait of eating disorders. Prevalence of BID is increasing as the anthropomorphic differences between ethnic and racial groups taper [29]. However, little research has been done to examine the prevalence, potential consequences, and correlates of BID among patients with mental illness in an Asian context. This study was designed to establish the prevalence of body image dissatisfaction (BID) among out-patients with mental illness in a tertiary care psychiatric hospital in Singapore: specifically, among those with schizophrenia spectrum disorder and depressive disorders. This study also elucidates the associations of BID with socio-demographic factors, and emotional and physical well-being. 

## 2. Methodology

### 2.1. Sample and Procedures

An epidemiological, single-phase cross-sectional study was conducted for a period of two years to estimate the prevalence of BID. Participants for this study were recruited from an out-patient clinic at a tertiary care psychiatric hospital, the Institute of Mental Health (IMH) in Singapore. The inclusion criteria comprised English speaking patients aged 18–40 years old (youngest age group: 18 to 23 years old; younger age group: 24 to 29 years old; young age group: 30 to 35 years old; middle age group: 36 to 40 years old), belonging to Singapore’s multi-ethnic population: Chinese, Malay, Indian, and Others; diagnosed with either schizophrenia spectrum disorders or depressive disorders. The clinical diagnosis of the patients was determined as per International Classification of Diseases, 9th Revision (ICD-9R) criteria. Patient’s diagnoses were corroborated through their medical records and clinician diagnosis. The participants were recruited by being approached in the out-patients clinic as well as by being referred by the clinicians. The treating clinicians were aware about patient’s functioning levels from the cognition level assessments they do. Thus, when patients agreed to participate in current study, the study team member doing recruitment confirmed from the clinicians whether the patient was cognitively able to do the survey. Trained study team members explained the study procedures before obtaining written informed consent from both the participants and the guardians/legally acceptable representatives of those aged 18–20 years, as the age of majority is 21 years in Singapore. English speaking patients who were unable to give signed informed consent were not included in the study. Participants who were not interested in participating in the study were not approached by the study team. A total of 310 participants were enrolled in the study. Data was collected using the self-administered survey questionnaire which included various scales pertaining to eating behaviors, body image, physical activity, and alcohol behavior. Survey instruments in the English language were utilized as only English literate participants were recruited as per the eligibility criteria of the study. On completion of the survey, participants were reimbursed with Singapore Dollar (SGD) 30 as an inconvenience fee.

### 2.2. Ethical Requirements

All study procedures and materials were approved by the relevant ethics and institutional review boards: The National Healthcare Group (NHG) Domain Specific Review Board (DSRB) (ethical approval code:2015/00206) and the IMH Clinical Research Committee (CRC). 

### 2.3. Main Instruments

(i) Socio-demographic questionnaire: This consisted of questions pertaining to age, gender, ethnicity, education level, marital and employment status, and personal/household income.
(ii) Body Shape Questionnaire-8c (BSQ-8c) [30]: This is an 8-item, self-administered questionnaire. It is used to evaluate fear of putting on weight, feelings of low self-esteem because of one’s appearance, the desire to lose weight, and body dissatisfaction. Each item is measured on a 6-point Likert scale, ranging from Never-1 to Always-6. Four groups are developed based on summation scores: no concern with shape (less than 19), mild concern with shape (between19–25), moderate concern with shape (between 26–33), and marked concern with shape (more than 33). Cronbach’s alpha was 0.933.
(iii) Eating Attitudes Test-26 (EAT-26) [31]: This is a 26-item screening measure of global eating attitude towards food and is a useful measure to identify those at risk for an eating disorder. Participant’s scores are assessed in three main domains: (1) dieting, (2) bulimia and food preoccupation, and (3) oral control scales. Scores range between 0 and 75 and are determined on a 6-point Likert scale (Always, Usually, Often, Sometimes, Rarely, Never) with scores greater than 20 indicative of disordered eating attitudes. The EAT-26 has been shown to be a psychometrically sound instrument displaying high internal consistency (α = 0.90). In this study, a cut-off score of >19 was used to indicate at high risk of eating behaviors. Cronbach’s alpha was 0.871.
(iv) Binge Eating Scale (BES) [32]: This is a 16-item self-report questionnaire designed to identify behavioral and cognitive characteristics of binge eating on a 4-point scale. Based on the scores, BES screens for binge eating behavior by classifying into three different levels of severity: non-binging (scoring 17 and less), moderate binging (scores between 18–26), and severe binging (scoring 27 or more). Cronbach’s alpha was 0.830.
(v) Beck’s Anxiety Inventory (BAI) [33]: This is a 21-item self-report measure of severity of anxiety in adults and adolescents on a 4-point scale. The BAI assesses emotional, physiological, and cognitive symptoms of anxiety. Each item refers to one of four aspects of anxiety behavior: (1) subjective (e.g., “unable to relax”), (2) neurophysiologic (e.g., “numbness or tingling”), (3) autonomic (e.g., “feeling hot”), or (4) panic-related (e.g., “fear of losing control”). A total score is summed for all 21 items ranges between 0 (no anxiety) to 63 (severe anxiety). Cronbach’s alpha was 0.948.
(vi) Beck’s Depression Inventory (BDI) [34]: This is a 21-item self-report measure of depression in adults and adolescents on a 4-point scale. The BDI assesses symptoms corresponding to criteria for diagnosing depressive disorders such as hopelessness, irritability, and guilt, as well as physical symptoms such as fatigue. A total score is summed for all 21 items ranging between 0 (no depression) to 63 (severe depression). Cronbach’s alpha was 0.935.
(vii) Body Mass Index (BMI) [35]: Nutritional status was assessed by means of the body mass index (BMI = body mass/(height)^2^). Participants’ BMI was calculated using an ultrasonic height sensor and load cell (Avamech, model B1000). The BMI scores were categorized according to cut-off points established by the Obesity and Metabolic Unit in the Singapore General Hospital: those having a BMI score of 27.5 and above are at high risk for obesity, score of 23–27.4 are at moderate risk for obesity, 18.5–22.9 are at low risk, and scores below 18.5 were at risk for nutritional deficiency.
(viii) Alcohol Use Disorders Identification Test (AUDIT) (WHO) [36]: This is a 10-item measure to screen for individuals with excessive, hazardous, and harmful drinking habits to the point of illness. Domains include hazardous alcohol use, dependence symptoms, and harmful alcohol use. Scoring is based on responses from 0 to 4 on the frequency that an item occurred. Cronbach’s alpha was 0.824.

### 2.4. Statistical Analysis

Data analysis was conducted on IBM SPSS Statistics version 23 (IBM Corp. Armonk, NY, USA). Descriptive statistics were done where quantitative data were shown as mean and standard deviation (SD). The qualitative data were shown as the frequency and percentage to define the prevalence, variety of categories, and sub-groups that were identified in the study. BSQ-8c mean scores and standard deviation of 310 responses were calculated for each socio-demographic subgroup in order to compare differences. The associations of BID were established with the use of multivariable linear regressions. Two regression models were used to measure effect of sociodemographic factors, BAI, BDI, BES, EAT-26, and AUDIT severity levels on the body shape dissatisfaction (BSQ-8c): First model was based on independent effect of socio-demographic characteristics like gender, age groups, ethnicity, marital status, employment status, education level, diagnosis and BMI groups, and the second model was adjusted for the above mentioned variables along with BAI, BDI, BES, EAT-26, and AUDIT severity levels. Normality and collinearity among the variables were checked before running the regression analysis. All statistically significant results were reported at *p* ≤ 0.05. 

## 3. Results

Our study collected the data from 310 out-patients in a tertiary care psychiatric hospital in Singapore. The sample was equally distributed in terms of gender. Based on the summation scores on the BSQ-8c scale, the prevalence of BID observed was 30.9%. Among the participants, 16.1% had scores of moderate concern and 14.8% had scores with marked concern (Table 1). 

Females had higher mean scores of BSQ-8c (M = 24.23, SD = 10.26) compared to males (M = 20.29, SD = 9.50). The youngest group aged 18–23 years showed significantly higher mean scores (M = 23.41, SD = 11.43) compared to the other (younger, young, and middle) age groups. Among ethnic groups, the Indian ethnic group indicated the highest mean BSQ-8c score (M = 23.93, SD = 10.07) compared to other ethnicities (Table 2). Participants who completed university education had the highest BSQ-8c mean scores (M = 23.13, SD = 9.07) compared to those participants who completed polytechnic diplomas and primary or secondary education (Table 2). Among study participants, those who were homemakers, unemployed, or retired displayed highest BSQ-8c mean scores (M = 23.24, SD = 10.18) compared to other employment categories. Participants who were separated, divorced, or widowed had higher means scores of BSQ-8c (M = 24.05, SD = 7.29) than those who were married and who were never married or single (Table 2). Participants diagnosed with depressive disorders (M = 23.18, SD = 10.49) had higher BSQ-8c mean scores than those diagnosed with schizophrenia spectrum disorders (M = 21.34, SD = 9.58). Participants with high risk BMI scores (BMI > 27.5) had the highest means of BSQ scores (M = 26.31, SD = 9.70) (Table 2).

Correlates of body shape dissatisfaction were established through two regression models. The regression model 1 in Table 3 examined the associations of BSQ-8c while adjusting for socio-demographic variables. Females were more likely to have higher BSQ-8c scores (B= 4.621, *p* < 0.001) compared to males (Table 3, model 1). There were no significant associations seen between the body image dissatisfaction and age groups, ethnicities, education, marital status, and employment. Participants with high risk BMI scores (B = 4.103, *p* < 0.004) were more likely to be dissatisfied with their body shape compared to those with moderate risk (Table 3, model 1).

Regression model 2 adjusted for socio-demographic variables and categories from BAI, BDI, BES, EAT-26, and AUDIT. Model 1 differed from Model 2 as the positive association of being female and BID was not significant in Model 2. Model 2, similar to model 1, indicated that participants with high risk BMI scores were also significantly associated with BID (B = 4.148, *p* < 0.016) (Table 3, model 2). Participants showing severe levels of depression on the BDI scale (B = 5.182, *p* < 0.007) were more likely to endorse body shape dissatisfaction than those with low levels of depression. Based on the BES scale, participants who displayed moderate risk (B = 4.515, *p* < 0.028) and severe risk (B = 8.832, *p* < 0.004) were more likely to be dissatisfied with their body shape. The scores from the EAT-26 scale similarly indicated that participants at risk (B = 10.66, *p* < 0.001) were more likely to be dissatisfied with their body shape as compared to those at no risk. No significant associations were found between hazardous drinking, anxiety symptoms, and body shape dissatisfaction.

## 4. Discussion

The current study established prevalence and correlates of BID among psychiatric out-patients with depressive and schizophrenia spectrum disorders. The prevalence of BID observed in our sample is 30.9%. Of this, 14.8% patients expressed marked concerns with BID, which is lower compared to prior research conducted among American inpatient psychiatric populations which reported 22.1% of body image concerns [37]. The variation in prevalence of BID could be due to differences in the study samples and setting; in this case, the participants being out-patients may have higher levels of functioning as compared to in-patients. Most previous research has found a strong association between depression and weight gain [38] that may cause poor body image attributing to poor self-esteem, lack of social support, and suicidal ideation [39,40]. Participants diagnosed with schizophrenia may express lower degree of BID due to dysfunction of sensory information processing as compared to those diagnosed with depression [41]. 

There were no significant associations seen between the BDI and age groups, ethnicities, education, marital status, and employment in the current study. However, our study observed positive, significant associations between BID and females, risk of obesity, depression, and eating disorders. Females in our study showed significant association with BID over males—a finding that is consistent with prior research [42]. Females are more worried and dissatisfied with their body image than males due to the social pressure for a slim body and hence being overweight or even imagining oneself being overweight leads to a higher BID [43]. BID among females is related to self-perceptions of being overweight by evaluating specific body features negatively which may pose a risk for anxiety disorders [44,45], whereas males are more likely to be distressed with developing their upper body and being muscular [46,47,48]. Prior evidence also suggests that, while males tend to show dissatisfaction with slimness, being female is more likely to be associated with dissatisfaction due to excess weight [49,50]. This study identified a positive and significant association between obesity and BID. This finding is consistent with previous studies which suggested that higher body weight is seen as a stigma and is considered unattractive, leading to the development of BID in individuals with high risk BMI [3,51,52]. It has been observed that high risk BMI scores are positively related to BID, which may strongly influence the adoption and development of abnormal eating attitudes [53]. On the contrary, Bosi et al. [54] reported that there was no association seen between BID and BMI among 82.9% of participants who had normal weight and 11.4% who were overweight in their sample. 

Binge eating is known to be associated with various adverse outcomes like early onset of obesity, frequent weight fluctuations, psychiatric illnesses, and BID [55,56,57]. Results from our study also indicated BID to be strongly associated with moderate and severe levels of binge eating behavior. This data is in line with a previous study which suggests that BID is a risk factor for binge eating behavior [58]; other studies showed that BID is positively associated with binge eating behavior, low self-esteem, negative affect, depression, and suicidal ideation, which poses serious mental health concerns [59,60,61]. A study among African-Americans showed that BID, mood, and drive for thinness were main contributors for binging which were triggered by episodes of anxiety [62]. In line with prior research, the current study also found that BID is most likely to be associated with eating attitudes [63,64]. Increase in body weight is usually associated with increased BID, specifically body part dissatisfaction and early onset of abnormal eating behaviors [65,66] which may be attributed to societal pressures and media’s influence on the development and maintenance of eating and body image disorders [67]. Eating problems are noted to be highest among those with negative body image problems owing to failed dieting and depression [68]. Body shape and weight over-evaluation with impairments in psychological functioning exhibited a robust relationship on binge eating disorders [69,70]. On the contrary, another study reported that BID is not associated with eating attitudes and is present among adolescents irrespective of whether they exhibit behaviors that make them vulnerable to eating disorders [71]. 

BID is well identified as a risk factor for the development of depression, anxiety, and disordered eating [72]. A longitudinal study indicated that BID is a predisposing factor to various psychological disorders such as depressive disorder among different age groups [73]. Consistent with previous research, our study also found that depressive disorders were more likely to be associated with BID [74]. This finding may be attributed to distressing and impairing body image concerns that appear to be prevalent among individuals with depression, as BID and depressive disorders are often co-existing mental disorders [74]. Individuals diagnosed with depression are more likely to overeat, make poor food choices, and have a sedentary lifestyle which leads to obesity and poor body image [75].

The strengths of the study include that it was a single-site study with large sample size, conducted by trained researchers using structured instruments that included a multi-ethnic Asian population with psychiatric illnesses. The study also found that BID is higher among those with depressive disorders, but this finding needs further investigation. The results from the current study fill the gap in literature in relation to prevalence of BID and its risk factors among a psychiatric out- patient population. Furthermore, the study adds to the growing body of literature that states that the prevalence of BDI among psychiatric out-patients in Asian countries is lower than that reported in the West. The study exhibits the findings of associations of body image dissatisfaction with depression and anxiety among psychiatric out-patients while identifying some unique and intriguing aspects of binge eating, obesity, and eating disorders with BDI.

However, certain limitations need to be considered while interpreting the study findings. Being a cross-sectional study, we were unable to establish any temporal relationships between BID and its correlates. The study was conducted in a single, tertiary psychiatric institution, among those aged 18–40 years, and hence the results may not be generalizable to other clinical settings. There is a possibility of selection bias as a result of convenience sampling of English-speaking psychiatric out-patients. As the questionnaires were available only in English, we recruited only those who could read and understand English. Singapore has a high English literacy rate, as suggested by data from the 2015 General Household survey, which showed that English literacy among those aged 15 years and over was 83.1% (Department of Statistics, Singapore, 2015). As the cohort of patients recruited for this study were young, we did not encounter significant issues in terms of language ineligibility, but unfortunately, reasons for refusal and ineligibility were not captured and thus we are unable to provide accurate numbers of patients who could not speak English. Also, patients who refused to participate in this study could have been at higher risk of BID. We were unable to collect any data from those who refused to participate in the study, which again limits the generalizability of the findings. 

## 5. Conclusions

In conclusion, the current study reflects that BID is prevalent among the female psychiatric population in Singapore and is associated with high BMI, eating disorders, binge eating behavior, and depressive disorders. Further research is needed to investigate body image dissatisfaction in the study population, especially in terms of different age groups. Comorbidity is best treated when both conditions are tackled simultaneously. Hence, the first step towards it is that clinicians should be cognizant of the presence of BID in their patients through regular assessments and screening as body dissatisfaction may exacerbate behavioral problems and relapse. Awareness of prevalence of body image dissatisfaction and the magnitude of its effects on an individual’s behavior must be emphasized by clinicians to their patients. This will be beneficial in early detection and its treatment in order to prevent the increase in BID prevalence in this population.

## Figures and Tables

**Table 1:** Descriptive statistics and Classification of BID based on the BSQ-8c scores.

BSQ_CONCERN		Frequency	Percent	Valid Percent	Cumulative Percent
Valid	No concern	124	40	40	40
	Mild concern	90	29	29	69
	Moderate concern	50	16.1	16.1	85.2
	Marked concern	46	14.8	14.8	100
	Total	310	100	100	

**Table 2:** Socio-demographic characteristics and descriptive statistics for Body Shape Questionnaire (BSQ) scores, anxiety severity, depression severity, binge eating risk and eating attitude risk.

Variable		Sample		BSQ Score	
		N	Sample (%)	Mean	SD
**Gender**	Male	155	50	20.29	9.5
	Female	155	50	24.23	10.26
**Age (years)**	18–23 (youngest)	78	25.16	23.41	11.43
	24–29 (younger)	100	32.26	22.39	10.19
	30–35 (young)	97	31.29	21.04	8.3
	36–40 (middle)	35	11.29	22.71	11.01
**Ethnicity**	Chinese	218	66.26	21.99	9.64
	Malay	51	15.5	23.1	11.73
	Indian	29	8.81	23.93	10.07
	Others	12	3.65	19.58	10.59
**Education ^a^**	Secondary and below	18	5.49	21.67	11.17
	O/N/A Level	94	28.66	21.47	10.34
	ITE/Diploma/Other Professional Qualifications	137	41.77	22.46	10.24
	University	60	18.29	23.13	9.07
**Employment Status ^b^**	Employed	152	47.06	21.97	9.32
	Student/NS *	62	19.2	21.66	11.39
	Unemployed/retired/homemaker	90	27.86	23.24	10.18
**Marital Status**	Single/never married	259	78.72	21.97	10.01
	Married	32	9.73	23.56	11.92
	Divorced/Separated/Widowed	19	5.78	24.05	7.29
**Diagnosis**	Depression	154	46.81	23.19	10.49
	Schizophrenia	156	47.42	21.35	9.58
**BMI (SG)**	Risk of nutritional deficiency	31	9.42	18.97	11.06
	Low risk	90	27.36	19.19	9.17
	Moderate risk	99	30.09	22.4	9.66
	High risk	90	27.36	26.31	9.71
**Anxiety Severity**				17.19	13.51
**Depression Severity**				17.86	13.73
**Binge eating risk**				9.82	8.27
**Eating attitude risk**				8.94	8.87

N = 310, ^a^ *n*= 309, ^b^ *n* = 304. NS * (National Service)—A statutory requirement of compulsory period of military training in Singapore for all male Singaporean citizens and permanent residents attaining the age of 18.

**Table 3:** Associations between BSQ-8c scores and socio-demographic characteristics, body mass index (BMI), depressive and anxiety symptoms, binge eating behaviors, eating attitudes, and alcohol behavior.

Variable	Model 1			Model 2		
	B	*p* Value	(95% CI)	B	*p* Value	(95% CI)
**Gender**						
Female	4.621	0	(2.349, 6.893)	2.297	0.1	−0.443, 5.037
Male	1 (Reference)			1 (Reference)		
**Age**						
18–23 (Youngest)	3.099	0.074	(−0.306, 6.503)	2.502	0.227	(−1.576, 6.58)
30–35 (Young)	−2.057	0.138	(−4.783, 0.668)	−0.49	0.761	(−3.669, 2.688)
36–40 (Middle)	−2.283	0.25	(−6.184, 1.617)	−0.777	0.758	(−5.743, 4.19)
24–29 (Younger)	1 (Reference)			1 (Reference)		
**Ethnicity**						
Malay	0.806	0.586	(−2.105, 3.718)	−0.769	0.778	(−6.164, 4.625)
Indian	0.898	0.634	(−2.814, 4.609)	−0.378	0.887	(−5.62, 4.863)
Others	−3.158	0.261	(−8.68, 2.365)	−4.133	0.215	(−10.7, 2.434)
Chinese	1 (Reference)			1 (Reference)		
**Marital Status**						
Married	0.222	0.909	(−3.575, 4.018)	−1.986	0.445	(−7.116, 3.143)
Separated, Divorced or Widowed	1.452	0.547	(−3.287, 6.192)	1.418	0.578	(−3.608, 6.444)
Never married	1 (Reference)			1 (Reference)		
**Employment status**						
Student/NS	−1.42	0.445	(−5.075, 2.236)	1.059	0.62	(−3.157, 5.274)
Unemployed, Homemaker or Retired	1.334	0.307	(−1.233, 3.902)	2.044	0.196	(−1.066, 5.155)
Employed	1 (Reference)			1 (Reference)		
**Education Level**						
PSLE/Secondary	−0.374	0.877	(−5.123, 4.375)	−1.947	0.498	(−7.617, 3.723)
O/N/A Levels	−0.053	0.968	(−2.643, 2.538)	−0.765	0.626	(−3.863, 2.332)
University	0.692	0.651	(−2.32, 3.704)	1.262	0.471	(−2.187, 4.712)
ITE/Poly/Others	1 (Reference)			1 (Reference)		
**Diagnosis**						
Schizophrenia spectrum disorders	−2.89	0.02	(−5.329, −0.45)	1.43	0.41	(−1.989, 4.849)
Depressive disorders	1 (Reference)			1 (Reference)		
**BMI Groups**						
Risk of nutritional deficiency	−7.162	0	(−11.164, −3.16)	−4.319	0.062	(−8.857, 0.219)
Low risk	−4.078	0.004	(−6.84, −1.316)	−0.945	0.563	(−4.167, 2.277)
High risk	4.103	0.004	(1.316, 6.89)	4.148	0.016	(0.79, 7.506)
Moderate risk	1 (Reference)			1 (Reference)		
**Anxiety Severity Levels**						
Mild				−2.392	0.211	(−6.154, 1.37)
Moderate				−2.234	0.243	(−5.999, 1.53)
Severe				−0.998	0.645	(−5.269, 3.273)
Low				1 (Reference)		
**Depression Severity Levels**						
Mild				2.44	0.272	(−1.939, 6.819)
Moderate				3.973	0.055	(−0.088, 8.033)
Severe				5.182	0.007	(1.463, 8.9)
Low				1 (Reference)		
**Binge Eating Risk**						
Moderate				4.515	0.028	(0.5, 8.53)
Severe				8.832	0.004	(2.837, 14.826)
Low				1 (Reference)		
**Eating Attitude Risk**						
At Risk				10.666	0	(6.179, 15.152)
No risk				1 (Reference)		
**Severity of Alcohol behaviour (AUDIT)**						
Moderate				2.361	0.198	(−1.249, 5.971)
High				−2.69	0.494	(−10.443, 5.062)
Very High				3.775	0.422	(−5.484, 13.034)
Low				1 (Reference)		

## References

[1] Xu X., Mellor D., Kiehne M., Ricciardelli L.A., McCabe M.P., Xu Y. (2010). Body dissatisfaction, engagement in body change behaviors and sociocultural influences on body image among Chinese adolescents. Body Image.

[2] Derenne J.L., Beresin E.V. (2002). Risk factors for body dissatisfaction in adolescent girls: A longitudinal investigation. Body image, media, and eating disorders. Dev. Psychol..

[3] Ferrari E.P., Petroski E.L., Silva D.A.S. (2013). Prevalence of body image dissatisfaction and associated factors among physical education students. Trends Psychiatry Psychother..

[4] Petroski E.L., Pelegrini A., Glaner M.F. (2012). Reasons and prevalence of body image dissatisfaction in adolescents. Cien Saude Colet.

[5] Pelegrini A., Coqueiro R.S., Beck C.C., Ghedin K.D., Lopes A.S., Petroski E.L. (2014). Dissatisfaction with body image among adolescent students: Association with socio-demographic factors and nutritional status. Cien Saude Colet.

[6] Santana M.L., Silva Rde C., Assis A.M., Raich R.M., Machado M.E., de JPinto E., de Moraes L.T., Ribeiro Júnior Hda C. (2013). Factors associated with body image dissatisfaction among adolescents in public schools students in Salvador, Brazil. Nutr. Hosp..

[7] As-Sa’edi E., Sheerah S., Al-Ayoubi R., Al-Jehani A., Tajaddin W., Habeeb H. (2013). Body image dissatisfaction: Prevalence and relation to body mass index among female medical students in Taibah University, 2011. J. Taibah Univ. Med. Sci..

[8] Steg L., Buunk A.P., Rothengatter T. (2008). Applied Social Psychology: Understanding and Managing Social Problems.

[9] Paxton S.J., Neumark-Sztainer D., Hannan P.J. (2006). Body dissatisfaction prospectively predicts depressive mood and low self-esteem in adolescent girls and boys. J. Clin. Child Adolesc. Psychol..

[10] Toro J., Gomez-Peresmitre G., Sentis J. (2006). Eating disorders and body image in Spanish and Mexican female adolescents. Soc. Psychiatry Psychiatr. Epidemiol..

[11] Shin N.Y., Shin M.S. (2008). Body dissatisfaction, self-esteem and depression in obese Korean children. J. Pediatr..

[12] Shepherd H., Ricciardelli L.A. (1998). Test of Stice’s dual pathway model: Dietary restraint and negative affect as mediators of bulimic behavior. Behav. Res. Ther..

[13] Rodriguez-Cano T., Beato-Fernandez L., Llario A.B. (2006). Body dissatisfaction as a predictor of self-reported suicide attempts in adolescents: A Spanish community prospective study. J. Adolesc. Health.

[14] Brausch A.M., Muehlenkamp J.J. (2007). Body image and suicidal ideation in adolescents. Body Image.

[15] Archibald K. (2010). The Role of Body Image and Social Anxiety in Problematic Drinking Behavior. Undergrad. Rev..

[16] Olivardia R., Pope H.G., Borowiecki J.J., Cohane G.H. (2004). Biceps and Body Image: The Relationship Between Muscularity and Self-Esteem, Depression, and Eating Disorder Symptoms. Psychol. Men Masc..

[17] Ali M.M., Fang H., Rizzo J.A. (2010). Body weight, self-perception and mental health outcomes among adolescents. J. Ment. Health Policy Econ..

[18] Goldfield G.S., Moore C., Henderson K., Buchholz A., Obeid N., Flament M.F. (2010). Body dissatisfaction, dietary restraint, depression, and weight status in adolescents. J. Sch. Health.

[19] Sertoz O.O., Doganavsargil O., Elbi H. (2009). Body image and self-esteem in somatizing patients. Psychiatry Clin. Neurosci..

[20] Kostanski M., Gullone E. (1998). Adolescent body image dissatisfaction: Relationships with self-esteem, anxiety, and depression controlling for body mass. J. Child Psychol. Psychiatry.

[21] Arbour-Nicitopoulos K.P., Faulkner G.E., Cohn T.A. (2010). Body image in individuals with schizophrenia: Examination of the B-WISE (R) questionnaire. Schizophr. Res..

[22] Oh E., Song E., Shin J. (2017). Individual factors affecting self-esteem, and relationships among self-esteem, body mass index, and body image in patients with schizophrenia. Arch. Psychiatr. Nurs..

[23] Yanhui L., Natalie P., Knoesenb C., David J., Castleb C., Jinsong T., Yunlong D., Riteesh B., Xiaogang C., Wei H. (2010). Symptoms of disordered eating, body shape, and mood concerns in male and female Chinese medical students. Compr. Psychiatry.

[24] Hayashi F., Takimoto H., Yoshita K. (2006). Perceived body size and desire for thinness of young Japanese women: A population-based survey. Br. J. Nutr..

[25] Makino M., Hashizume M., Yasushi M. (2006). Factors associated with abnormal eating attitudes among female college students in Japan. Arch Women Ment. Health.

[26] Boon E., Zainal K.A., Touyz S.W. (2017). Perceptions of eating disorder diagnoses and body image issues in four male cases in Singapore. J. Eat. Disord..

[27] Jiang W., Tan J., Fassnacht D.B. (2017). Implicit and explicit anti-fat bias among Asian females. Eat. Weight Disord..

[28] Luo Y., Parish W.L., Laumann E.O. (2005). A population-based study of body image concerns among urban Chinese adults. Body Image.

[29] Spigarelli M.G., Haney S.E., Butler K., Sotiriou E.G. (2012). Assessing Body Dissatisfaction in Medical Students: Results of the BSQ-8C (Body Shape Questionnaire). J. Adolesc. Health.

[30] Cooper P.J., Taylor M.J., Cooper Z., Fairbum C.G. (1987). The development and validation of the Body Shape Questionnaire. Int. J. Eat. Disord..

[31] Garner D.M., Olmsted M.P., Bohr Y. (1982). The eating attitudes test: Psychometric features and clinical correlates. Psychol. Med..

[32] Gormally J., Black S., Daston S., Rardin D. (1982). The assessment of binge eating severity among obese persons. Addict. Behav..

[33] Beck A.T., Epstein N., Brown G., Steer R.A. (1988). An inventory for measuring clinical anxiety: Psychometric properties. J. Consult. Clin. Psychol..

[34] Beck A.T., Beck R.W. (1972). Screening depressed patients in family practice. A rapid technic. Postgrad. Med..

[35] Singapore General Hospital, 2016 Obesity. http://www.sgh.com.sg/subsites/lifecentre/life%20centre%20specialties/units/obesity-and-metabolic-unit/conditions-and-treatments/pages/obesity.aspx.

[36] World Health Organization Department of Mental Health and Substance Dependence The Alcohol Use Disorders Identification Test. Guidelines for Use in Primary Care. Second Edition. http://apps.who.int/iris/bitstream/10665/67205/1/WHO_MSD_MSB_01.6a.pdf.

[37] Jennifer D., Jennifer K., Katharine A.P., Jeffrey I.H. (2006). Body Dysmorphic Disorder and Other Clinically Significant Body Image Concerns in Adolescent Psychiatric Inpatients: Prevalence and Clinical Characteristics. Child Psychiatry Hum. Dev..

[38] Gregory E.S., Michael V.K., Kathleen S., Diana L.M., Paul K.C., Gerald B., Ronald C.K. (2006). Association Between Obesity and Psychiatric Disorders in the US Adult Population. Arch Gen. Psychiatry.

[39] Jacqueline A.P., Thomas R.S., Elizabeth J. (2000). Psychosocial differences associated with body weight among female adolescents: The importance of body image. J. Adolesc. Health.

[40] Brechan I., Kvalem I.L. (2015). Relationship between body dissatisfaction and disordered eating: Mediating role of self-esteem and depression. Eat. Behav..

[41] Stefan P., Frank R. (2001). Specific body image pathology in acute schizophrenia. Psychiatry Res..

[42] Thomas J., Khan S., Abdul Rahman A.A. (2010). Eating attitudes and body image concerns among female university students in the United Arab Emirates. Appetite.

[43] Espina M., Ortego A., de Alda O., Aleman A., Juaniz M. (2002). Body shape and Eating disorders in a sample of students in the Basque country: A pilot Study. Psychol. Spain.

[44] Seid R.P. (1989). Never too Thin: Why Women Are at War with Their Bodies.

[45] Heatherton T.F., Hebl M.R., Friedman H.S. (1998). Body Image. Encyclopedia of Mental Health.

[46] Geoffrey H.C., Pope H.G. (2001). Body image in boys: A review of the literature. Int. J. Eat. Disord..

[47] Rheanna N.A., Alison B.L., Megan M.L. (2007). The Effects of Gender and Family, Friend, and Media Influences on Eating Behaviors and Body Image During Adolescence. J. Youth Adolesc..

[48] McCabe M.P., Ricciardelli L.A. (2004). Body image dissatisfaction among males across the lifespan: A review of past literature. J. Psychosom. Res..

[49] Cassiano R., Eliane D.S., Joyce D.R. (2010). Self-perception of body image in the physical education course students. Rev. Bras. Educ. Fís. Esporte São Paulo.

[50] Silva T.R., Guilherme S., Érico F.P., Motriz R.C. (2011). Factors associated with body image in Physical Education students. Motriz: Revista Educação Física.

[51] Idalina S.K., Sebastião A. (2006). Relationship between body mass index and self-perception among university students. Rev. Saúde Pública.

[52] Jaworowska A., Bazylak G. (2009). An outbreak of body weight dissatisfaction associated with self-perceived BMI and dieting among female pharmacy students. Biomed. Pharmacother..

[53] Calzo J.P., Sonneville K.R., Haines J., Blood E.A., Field A.E., Austin S.B. (2012). The development of associations among body mass index, body dissatisfaction, and weight and shape concern in adolescent boys and girls. J. Adolesc. Health.

[54] Bosi M.L.M., Luiz R.R., Morgado C.M.C., Carvalho R.J. (2006). Self-perception of body image among nutrition students: A study in the city of Rio de Janeiro. J. Bras. Psiquiatr..

[55] Yanovski S.Z. (1993). Binge eating disorder: Current knowledge and future directions. Obes. Res..

[56] Ivezaj V., Saules K.K., Hoodin F., Alschuler K., Angelella N.E., Collings A.S., Saunders-Scott D., Wiedemann A.A. (2010). The relationship between binge eating and weight status on depression, anxiety, and body image among a diverse college sample: A focus on Bi/Multiracial women. Eat. Behav..

[57] Wardle J., Waller J., Rapoport L. (2001). Body dissatisfaction and binge eating in obese women: The role of restraint and depression. Obes. Res..

[58] Lofrano-Prado M.C., Prado W.L., Barros MV G., De Souza S.L. (2015). Eating disorders and body image dissatisfaction among college students. ConScientiae Saúde.

[59] Mori K., Sekine M., Yamagami T., Kagamimori S. (2009). Relationship between body image and lifestyle factors in Japanese adolescent girls. Pediatr. Int..

[60] Cargill B.R., Clark M.M., Pera V., Niaura R.S., Abrams D.B. (1999). Binge eating, body image, depression, and self-efficacy in an obese clinical population. Obes. Res..

[61] Tan E.S.L., Hawkins R.M.F. (2017). Psychological and behavioural characteristics of females with anorexia nervosa in Singapore. Eat. Weight Disord..

[62] Napolitano M.A., Himes S. (2011). Race, weight, and correlates of binge eating in female college students. Eat. Behav..

[63] Alves E., Vasconcelos F., Calvo M., Neves J. (2008). Prevalence of symptoms of anorexia nervosa and dissatisfaction with body image among female adolescents in Florianópolis, Santa Catarina State, Brazil. Cadernos Saúde Pública.

[64] Cheah W.L., Hazmi H., Chang C.T. (2015). Disordered eating and body image issues and their associated factors among adolescents in urban secondary schools in Sarawak, Malaysia. Int. J. Adolesc. Med. Health.

[65] Levine M.P., Smolak M., Cash T.F., Pruzinsky T. (2002). Body image development in adolescence. Body Image: A Handbook of Theory, Research, and Clinical Practice.

[66] Koff E., Rierdan J. (1993). Advanced pubertal development and eating disturbance in early adolescent girls. J. Adolesc. Health.

[67] Kevin T., Leslie J.H. (1999). The Media’s Influence on Body Image Disturbance and Eating Disorders: We’ve Reviled Them, Now Can We Rehabilitate Them? Version of Record online: 17 DEC 2002. J. Soc. Issues.

[68] Koenig L.J., Wasserman E.L. (1995). Body image and dieting failure in college men and women: Examining links between depression and eating problems. Sex. Roles.

[69] Linardon J. (2016). Correlates of the over-evaluation of weight and shape in binge eating disorder and mixed eating disorder samples: A meta- analytic review. Eat. Disord..

[70] Linardon J., Phillipou A., Castle D., Newton R., Philippa H., Cistullo L.L., Griffiths S., Hindle A., Brennan L. (2018). The relative associations of shape and weight over-evaluation, preoccupation, dissatisfaction, and fear of weight gain with measures of psychopathology: An extension study in individuals with anorexia nervosa. Eat. Behav/.

[71] Martins C.R., Pelegrini A., Matheus S.C., Petroski E.L. (2010). Body image dissatisfaction and its relationship with nutritional status, body fat, and anorexia and bulimia symptoms in adolescents. Rev. Psiquiatr. Rio Gd. Sul.

[72] Silberg J.L., Bulik C.M. (2005). The developmental association between eating disorders symptoms and symptoms of depression and anxiety in juvenile twin girls. J. Child. Psychol. Psychiatry.

[73] Nemark–Sztainer D., Paxton S.J., Hannan P.J., Haines J., Story M. (2006). Does body satisfaction matter? Five-year longitudinal associations between body satisfaction and health behaviors in adolescent females and males. J. Adolesc. Health.

[74] Schulte S.J., Thomas J. (2013). Relationship between eating pathology, body dissatisfaction and depressive symptoms among male and female adolescents in the United Arab Emirates. Eat. Behav..

[75] James D. Poor Body Image and Depression. https://www.eatingdisorderhope.com/information/body-image/poor-body-image-and-depression.

